# Predictive Factors of Intestinal Necrosis in Acute Mesenteric Ischemia

**DOI:** 10.1515/med-2019-0104

**Published:** 2019-12-17

**Authors:** Alfonso Canfora, Antonio Ferronetti, Gianpaolo Marte, Vittorio Di Maio, Claudio Mauriello, Pietro Maida, Vincenzo Bottino, Giovanni Aprea, Bruno Amato

**Affiliations:** 1 Department of General Surgery, Evangelic Hospital Betania, Via Argine, 604 - 80147, Naples-Italy; 2 Department of General Surgery, University of Naples Federico II, Naples, Italy

**Keywords:** Acute mesenteric ischemia, Irreversible transmural intestinal necrosis, Explorative laparotomy, Predictive factors

## Abstract

**Objectives:**

Acute mesenteric ischemia (AMI) is a gastrointestinal and vascular emergency in which the detection of patients requiring intestinal resection is mandatory.

**Methods:**

Registered data of 55 consecutive patients admitted to our center between January 2010 and December 2016 that underwent an explorative laparotomy for a suspected diagnosis of irreversible transmural intestinal necrosis (ITIN) were analyzed. Demographic, clinical, laboratory and CT findings were statistically analyzed in order to search predictive factors of ITIN and their correlation to its clinical spectre.

**Results:**

Tobacco use was the most statistically significant (p<0.01) cardiovascular disease risk factor involved in ITIN. Among lab tests, Serum lactate levels ˃ 2mmol/L resulted in a statistically significant association with ITIN (p=0.0001). Organ failure (defined as Marshall score> 2) and the three main CT findings (decreased bowel wall enhancement, bowel loop dilation and demonstrated vessel occlusion) were strongly associated with ITIN (p values: 0.001, 0.007, 0.0013, 0.0005). Only serum lactate levels>2 mmol/L resulted as statistically significant as predictive factors of ITIN in multivariate analysis using logistic regression (OR 49.66 and p-value 0.0021).

**Conclusion:**

Our univariate and multivariate analysis identified multiple factors (Serum lactate levels ˃ 2mmol/L, Organ failure, CT signs) that could suggest patients that require a surgical approach for ITIN.

## 1 Introduction

Acute mesenteric ischemia (AMI) is a gastrointestinal and vascular emergency, with an incidence that increases exponentially with age [1]. The short-term mortality of AMI is high and is related to the development of intestinal necrosis, which often brings surgeons to extensive intestinal resections, and condemns patients who survive to short bowel syndrome [2].

A multimodal and multidisciplinary management of AMI in specialized intestinal stroke centers could decrease the rate of intestinal resection, preventing the progression from reversible to irreversible intestinal ischemic injury and improving survival. In the places where a stroke center is not available, the primary goal is the detection of patients requiring intestinal resection and that can survive to a surgical intervention [2].

The purpose of this retrospective study was to find a correlation between clinical, laboratory and CT findings in our study populations and early predictive factors of irreversible transmural intestinal necrosis (ITIN). The secondary goal was to evaluate possible predictive factors of unresectable massive ITIN and of cases that could be better managed in stroke units.

## 2 Methods

The study was approved by the local ethics committee and complied with the Declaration of Helsinki, in line with the Guidelines for Good Clinical Practice. All patients provided written informed consent before entering the study, with respect to study participation, and confidentiality statement of data collection according to Italian privacy policy.

### 2.1 Patient selection and primary outcome

All registered data of patients who were admitted to our center, Department of General Surgery- Evangelic Hospital Betania, between January 2010 and December 2016, that underwent an explorative laparotomy for a suspected diagnosis of ITIN were analyzed. Suspicion of ITIN was identified in cardiovascular risk-factor positive patients with either clinical findings (gastrointestinal hemorrhage and abdominal guarding), laboratory findings (organ failure, white blood cells (WBC) > 10^4^/ml, serum lactate levels >2mmol/l, increased LDH levels and c reactive protein (CRP) levels >50) and CT-imaging characteristics of AMI (bowel wall thickening/thinning, decreased bowel wall enhancement, bowel loop dilation, demonstrated vessel occlusion and pneumatosis intestinalis) (Table 1 and 2) [2]. Patients who didn’t undergo a preoperative CT-scan and/or whose CT images were not able to be reviewed were excluded from the study.

**Table 1:** Overall demographics and clinical presentation

	ITIN n=36 (%)	No ITIN n=19 (%)	Overall *n*=55 (%)	*P value/Chi-square*
Age (years)	61	56	59 (54–87)	0.053/1.64 (t-test)
BMI	29	27	28 (17–33)	0.087/1.37 (t-test)
Tobacco use	22 (61)	4 (21)	26 (47)	**0.0046**/8.006
Arterial hypertension	15 (42)	6 (32)	21 (38)	0.46/0.536
Dyslipidemia	10 (28)	3 (16)	13 (24)	0.319/0.99
Diabetes mellitus	8 (22)	1 (5)	9 (16)	0.106/2.614
History of cardiovascular disease	*9(25)*	*4 (21)*	*13 (24)*	0.743/0.107

*Clinical presentation*				
Gastrointestinal hemorrhage	2 (4)	1 (5)	(6)	0.964/0.002
Abdominal guarding	26 (72)	15 (79)	41 (75)	0.586/0.296
Organ failure	20 (56)	2 (11)	21 (38)	**0.001**/10.57
WBC > 10^4^/Ml	28 (78)	16 (84)	44 (80)	0.57/0.321
Serum lactate levels > 2mmol/lc	30 (83)	1 (5)	31 (56)	**0.00001**/30.819
LDH levels >	33 (92)	17 (89)	50 (91)	0.788/0.072
CRP levels > 50	25 (69)	15 (79)	40 (73)	0.452/0.566

**Table 2:** Overall CT signs univariate analysis

	ITIN *n*=36 (%)	No ITIN *n*=19 (%)	Overall *n*=55 (%)	*P value/Chi-square*
Bowel wall thickening/thinning	22 (61)	14 (74)	36 (65)	0.351/0.869
Decreased bowel wall enhancement	25 (69)	6 (32)	31 (56)	**0.007**/7.249
Bowel loop dilation	24 (67)	4 (21)	28 (51)	**0.0013**/10.353
Demonstrated Vessel occlusion	19 (53)	1 (5)	20 (36)	**0.0005**/12.133
Pneumatosis intestinalis	2 (6)	1 (5)	3 (5)	0.964/0.002

The primary outcome was the correlation between preoperative laboratory, clinical and CT findings with the presence/absence of ITIN in patients that underwent surgery. The ITIN cases were also substaged in massive (less than 50 cm left), submassive (more than 50 cm left) and partial (less than 100 cm involved), in terms of small bowel length involved.

The secondary outcome was the determination of predictive factors, among those that were analyzed for the primary outcome, of a useless laparotomy because of a massive ITIN and of cases that could have received a better workup in stroke units.

### 2.2 Data collection

Patients’ data were retrospectively analyzed in order to collect demographic, clinical, biological and radiological findings. Clinical variables such as gender, age, Body Mass Index (BMI), cardiovascular risk factors (i.e. diabetes mellitus, tobacco use, arterial hypertension, dyslipidemia), previous cardiovascular disease (myocardial infarction, cerebral ischemia, limb ischemia, Pulmonary embolism, Venous thromboembolism) and clinical presentation (abdominal guarding, GI bleeding, organ failure, MOF) were recorded.

Organ failure and multiple organ failure were assessed by Marshal scores ≥ 2 in any system or ≥ 6 overall respectively[3]. The following Lab parameters were charted: WBC, CRP serum levels, LDH serum levels and lactate serum levels. All preoperative CT-scans were reviewed by random radiologists that were blinded to surgical outcome. Researched radiological signs includedvessel occlusion, decreased bowel enhancement, pneumatosis intestinalis, bowel loop dilation, and fluid collection.

### 2.3 Statistical analysis

Quantitative data were reported as medians and range. Continuous parameters were dichotomized using commonly accepted cut-off values. Qualitative data were reported as number and percentage of patients. A Chisquare test performed in univariate analysis searched for an association between the explored factors and the presence/extension of ITIN. All dependent variables associated with ITIN were tested in multivariate analysis using logistic regression. All analyses were perfomed using Medcalc ver 19.1. This study was performed in accordance to ethical standards of observational studies and reported according to the STROBE guidelines [4].

## 3 Results

### 3.1 Patient characteristics

**Figure 1:**
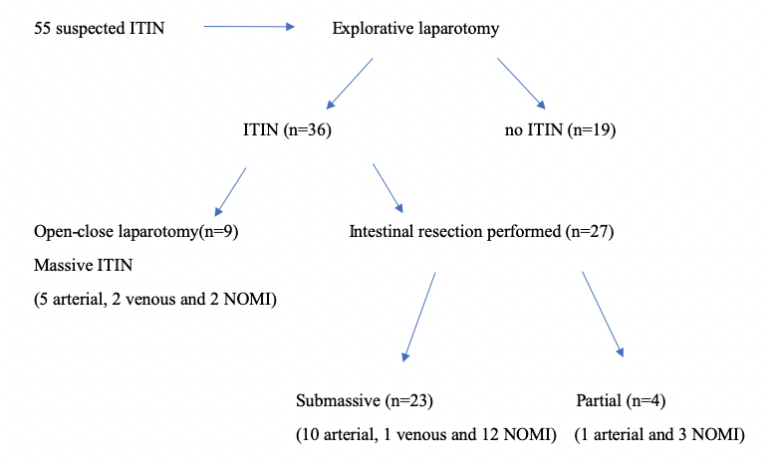
Flowchart of patients retrospectively analyzed

A total of 55 patients were consented to our study (Figure 1). 24 patients (44 %) were women; median age was 59 (54-87) years. Median BMI was 28 (17-33) (Table 1). Of the 55 patients who underwent an explorative laparotomy, 36 received a diagnosis of irreversible transmural intestinal necrosis (ITIN) while 19 received another diagnosis apart from irreversible acute mesenteric ischemia. Of the 36 patients receiving diagnosis of ITIN 9 were massive (5 arterial, 2 venous and 2 Non occlusive mesenteric ischemia-NOMI) and did not receive any resection, 23 were submassive (10 arterial, 1 venous and 12 NOMI) and 4 were partial (1 arterial and 3 NOMI) totaling 27 intestinal resections performed (Figure 1). The 19 patients with no ITIN received the following diagnosis: 7 intestinal volvuli, 3 colic diverticular perforations, 2 complicated Crohn diseases, 2 gastric perforations, 1 Meckel’s diverticulitis, 1 Meckel’s perforation, 1 occlusive ileal cancer and 2 no visceral alterations findings. Risk factors for cardiovascular disease (tobacco use, arterial hypertension, dyslipidemia, diabetes mellitus and history of cardiovascular disease) were present in both subgroups with a statistically significant difference only for tobacco use (p=0.0046), as shown in Table 1.

### 3.2 ITIN Clinical presentation

The most frequent clinical presentation was abdominal guarding but no statistical difference was noticed among the two subgroups. Inflammation lab testing such as CRP levels ˃50 and WBC ˃ 10^4^/ml were statistically non-specific for any of the two subgroups (Table 1). Concerning necrosis indexes, LDH levels were non-specific of ITIN, while Serum lactate levels ˃ 2mmol/L were statistically associated with ITIN (p=0.00001). Organ failure was also strongly associated with ITIN (p= 0.001) (Table 1) and statistically associated with massive ITIN(p=0.0201) (Table 3).

**Table 3:** Specific clinical and laboratory univariate analysis

	Massive ITIN *n*=9 (%)	Submassive/partial ITIN *n*=27 (%)	Overall *n*=36 (%)	*P value/Chi-square*
Gastrointestinal hemorrhage	1 (11)	1 (4)	2 (4)	0.4/0.705
Abdominal guarding	6 (67)	20 (74)	26 (72)	0.667/0.184
Organ failure-Marshall score≥6	8(89)	12 (44)	19 (53)	**0.0201**/5.4
WBC >10G/mL	6 (67)	22 (81)	28 (78)	0.355/0.857
Serum lactate levels >2mmol/mL	8 (89)	22 (81)	30 (83)	0.606/0.267
LDH levels >	9 (100)	24 (89)	33 (92)	N.A.
CRP levels > 50	6 (67)	19 (70)	25 (69)	0.835/0.043

### 3.3 ITIN CT presentation

Reviewing the 55 CT scans in the two subgroups, decreased bowel wall enhancement, bowel loop dilation and demonstrated vessel occlusion were the three researched CT signs to be statistically associated with ITIN (p values: 0.007, 0.0013, 0.0005) (Table 2 and 4). These three signs were non significantly associated with the various subgroups of ITIN analyzed. Bowel wall thickening/thinning and pneumatosis intestinalis were non-specific for ITIN detection.

**Table 4:** Specific CT-scan signs univariate analysis

	Massive ITIN *n*=9 (%)	Submassive/partial ITIN *n*=27 (%)	Overall *n*=36 (%)	P value/Chi-square
Bowel wall thickening/thinning	5 (56)	17 (63)	22 (61)	0.693/0.156
Decreased bowel wall enhancement	7 (78)	18 (67)	25 (69)	0.531/0.393
Bowel loop dilation	6 (67)	18 (67)	24 (67)	1/0
Demonstrated Vessel occlusion	7 (78)	12 (44)	19 (53)	0.083/3.01
Pneumatosis intestinalis	1 (11)	1 (4)	2 (6)	0.401/0.706

### 3.4 Multivariate analysis

Of the six statistically significant independent variables evaluated in univariate analysis, only serum lactate levels>2mmol/l were statistically significant as predictive factors of ITIN in multivariate analysis using logistic regression (OR 49.66 and p-value 0.0021) (Table 5).

**Table 5:** Independent variables multivariate analysis with logistic regression

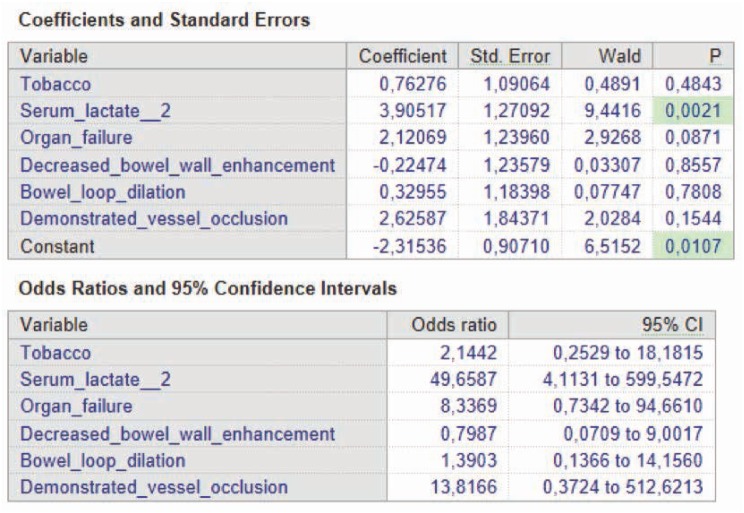

## 4 Discussion

Clinical presentation of AMI is often puzzling [1], especially in elderly patients, with cardiovascular risk factors or diseases. In our univariate analysis we identified predictive factors (Serum lactate levels ˃ 2mmol/ml, Organ failure, CT signs) (Table 1 and 3) that could suggest patients requiring a surgical exploration. Serum lactate levels [5], which generally decrease after surgical resection [6], and bowel loop dilatation CT signs [7] are strongly related with intestinal necrosis (Table 2 and 4). Contrary to common thought, abdominal guarding and pneumatosis intestinalis are not significantly associated with necrosis. Higher incidence of cardiovascular risk factors or comorbidities [8, 9] found in patients with ITIN is correlated with higher mortality. An earlier diagnosis of AMI, especially before the insurgence of multiorgan failure or clinical signs of peritonitis [10, 11], reduces morbidity and mortality, improving patients’ outcome and reducing surgical complications.

Cudnik et al. in 2013 with their systematic review and meta-analysis on the diagnosis of acute mesenteric ischemia , analyzing all diagnostic tests present in literature, concluded that signs, symptoms and laboratory findings are insufficiently diagnostic for acute mesenteric ischemia and that CT angiography is the only test with adequate accuracy (sensitivity 94% and specificity 95%) to establish diagnosis in lieu of laparotomy[12].

Our findings are in accordance with the results of Emile’s study [13] on the validity of CT vessel occlusion demonstration and acidosis (analyzed in our study as serum lactate levels) as predictive factors of ITIN; in our study leukocytosis was not statistically associated with ITIN probably due to different diagnosis timing or complication occurrence.

However, a recent meta-analysis by Treskes et al. has highlighted the possibility of introducing novel serological biomarkers to detect acute mesenteric ischemia. The intestinal fatty acid-binding protein (I-FABP), α-glutathione S-transferase (α-GST), ischemia modified albumin (IMA) and citrulline have demonstrated good sensitivity and specificity in the diagnosis of acute mesenteric ischemia but further research is still necessary to specify threshold values and accuracy standards for different aetiological forms [14].

Moreover, another recent systematic review by Khan et al. [15] on the diagnostic accuracy of classical and newly reported hematological parameters for AMI concludes that parameters such as Mean Platelet Volume (MPV), Neutrophil to Lymphocyte Ratio (NLR), and Red Cell Distribution Width (RDW) showed higher specificities than classical markers for diagnosis of AMI.

Despite research on novel treatment strategies for acute mesenteric ischemia, in hospital the mortality rate has not changed in the last decade, as shown in Adaba et al. recent study. Patients with arterial mesenteric infarction or with non-occlusive mesenteric infarction (NOMI) have a three times higher in-hospital mortality rate if compared with those with venous mesenteric infarction [16]. Better chances in terms of mortality and morbidity for patients with arterial occlusive AMI are offered by endovascular revascularization strategies [17].

In our experience, an accurate screening of patients that require surgery remains a challenge. CT-scanning is probably the best choice to find ITIN-suggesting features, in spite of its limits [12]. Although several studies tried to identify predictive factors related with ITIN, one recent study by Nuzzo et al [2] reports an association between organ failure, serum lactate levels (>2 mmol/L) and CT-scan bowel dilatation and ITIN, with a rate that increases depending on the presence of one or more of these factors. This study is one of the first that evaluates the need of an intestinal resection in the case of ITIN diagnosis. According to this study, the first aim of our work was to select patients with ITIN, who can benefit from a surgical intervention, avoiding unnecessary explorative laparotomies. Identifying specific predictive factors will help surgeons in the daily management of these critical patients.

This study has the limit of being a single centre study with a restricted cohort but brings in evidence the existence of predictive factors of ITIN that need to be further validated in wider and randomized studies.

## 5 Conclusion

The presence of at least one of the predictive lab/CT findings in high risk patients should dictate if an explorative laparotomy is mandatory because of the high risk of ITIN requiring resection. The presence of organ failure in addition to the precited risk factors of ITIN predicts a high risk of useless laparotomy for massive ITIN. Further studies should be designed in order to assess which patients should be treated in stroke centers.

## Abbreviation

AMI =: Acute mesenteric ischemia;
ITIN=: Irreversible transmural intestinal necrosis;
MOF=: Multiple organ failure;
NOMI=: Non occlusive mesenteric ischemia;
BMI =: Body mass index
